# Antibiotic Resistance of *Acinetobacter* spp. Isolates from the River Danube: Susceptibility Stays High

**DOI:** 10.3390/ijerph15010052

**Published:** 2017-12-30

**Authors:** Clemens Kittinger, Alexander Kirschner, Michaela Lipp, Rita Baumert, Franz Mascher, Andreas H. Farnleitner, Gernot E. Zarfel

**Affiliations:** 1 Institute of Hygiene, Microbiology and Environmental Medicine, Medical University Graz, Neue Stiftingtalstraße 2, 8010 Graz, Austria; Clemens.kittinger@medunigraz.at (C.K.); michaela.lipp@medunigraz.at (M.L.); rita.baumert@medunigraz.at (R.B.); franz.mascher@medunigraz.at (F.M.); 2 Institute for Hygiene and Applied Immunology, Water Hygiene, Medical University of Vienna, 1090 Vienna, Austria; alexander.kirschner@meduniwien.ac.at; 3 Interuniversity Cooperation Centre for Water and Health, Vienna University of Technology, 1060 Vienna, Austria; andreas.farnleitner@wavenet.at; 4 Institute of Chemical Engineering, Research Group Environmental Microbiology and Molecular Ecology, Vienna University of Technology, 1060 Vienna, Austria; 5 Karl Landsteiner University for Health Sciences, 3500 Krems, Austria

**Keywords:** *Acinetobacter*, JDS3, river, water, carbapenemases

## Abstract

*Acinetobacter* spp. occur naturally in many different habitats, including food, soil, and surface waters. In clinical settings, *Acinetobacter* poses an increasing health problem, causing infections with limited to no antibiotic therapeutic options left. The presence of human generated multidrug resistant strains is well documented but the extent to how widely they are distributed within the *Acinetobacter* population is unknown. In this study, *Acinetobacter* spp. were isolated from water samples at 14 sites of the whole course of the river Danube. Susceptibility testing was carried out for 14 clinically relevant antibiotics from six different antibiotic classes. Isolates showing a carbapenem resistance phenotype were screened with PCR and sequencing for the underlying resistance mechanism of carbapenem resistance. From the Danube river water, 262 *Acinetobacter* were isolated, the most common species was *Acinetobacter baumannii* with 135 isolates. Carbapenem and multiresistant isolates were rare but one isolate could be found which was only susceptible to colistin. The genetic background of carbapenem resistance was mostly based on typical *Acinetobacter* OXA enzymes but also on VIM-2. The population of *Acinetobacter* (*baumannii* and non*-baumannii*) revealed a significant proportion of human-generated antibiotic resistance and multiresistance, but the majority of the isolates stayed susceptible to most of the tested antibiotics.

## 1. Introduction

The genus *Acinetobacter* consists of over 40 known species that can be isolated from various habitats including soil, sediment surface, and wastewater [1]. They have the ability to colonize human skin and are responsible for a growing number of nosocomial outbreaks worldwide. Although most *Acinetobacter* species have generally a low pathogenicity [2], according to Alsan et al. (2008), the intensive care unit (ICU) mortality rate is around 40% [3].

The most striking characteristic of *Acinetobacter* spp. is their natural resistance to many antibiotics and the ability to easily develop new resistances under antibiotic pressure. They overexpress efflux pumps, harbor β-lactamases, and are characterized by low membrane permeability [2]. By 2012, over 210 different β-lactamases have been identified within the genus [4]. Different *Oxacillinases* (OXA) enzyme families have their origin in *Acinetobacter*, such as OXA-21like, OXA-23like or OXA-51like [5]. These enzymes are serine hydrolases represent class D according to the Ambler classification of β-lactamases [6]. The spread of these *Acinetobacter oxaxilinases* into other species seems much more limited than, for example, the spread of CTX-M or NDM enzymes but is documented. This set of OXA enzyme enables *Acinetobacter* to adapt easily to new developed β-lactam antibiotics [4,7]. Therefore, *Acinetobacter baumannii* especially has become one of the problematic nosocomial pathogens. Infections with some of these strains, such as bloodstream infections and pneumonia, do not leave any further options for antibiotic treatment. Next to *Pseudomonas* and carbapenem-resistant *Enterobacteriaceae*, *Acinetobacter* were rated by the WHO to be the group in most urgent need of new antibiotics (http://www.who.int/medicines/publications/ global-priority-list-antibiotic-resistant-bacteria/en/) [5,7,8]. In addition to the acquisition of a seemingly infinite number of resistances, such as *Pseudomonas* spp., *Acinetobacter* is characterized by a much better ability to survive hostile conditions, e.g., survival on dry surfaces. This makes *Acinetobacter* an ideal candidate for survival in clinical settings and in the environment [9,10,11]. 

Occurrence and susceptibility of *Acinetobacter* spp. in clinical settings is documented quite well, whereas their distribution and proportion of resistance in the aquatic environment remains quite unclear. Nearly all studies that investigate antibiotic resistance of *Acinetobacter* in the environment are based on selective cultivation, masking their proportion in the population, or are based on molecular methods, with all their inherent methodological weaknesses [9,12,13,14,15]. There is some evidence that environmental transport of *Acinetobacter* plays a role in the spread of clinical relevant *Acinetobacter* strains in the environment. On the other hand, there seems to be a continuous influx of novel strains into the clinical setting with the potential of new infectious features [16,17].

Participation in the Joint Danube Survey 2013 (JDS3) offered the possibility of isolating *Acinetobacter* from the total course of one of Europe’s longest rivers. This chance was taken to generate an initial picture of the resistance proportion within *Acinetobacter* spp. and to get an idea of how far acquired antibiotic resistances of clinical relevance have spread in the aquatic environment. 

## 2. .Material and Methods

### 2.1. Sample Collection

All samples were taken during the research expedition of the Joint Danube Survey 2013 (JDS3). The survey was organized by the International Commission for the Protection of the Danube River (ICPDR), Vienna. The water samples were taken between 12 August and 26 September 2013, from 68 sampling sites along the River Danube, starting at Böfinger Halde (Germany) downstream to the delta (Romania). At each sampling site, samples were collected at three sampling points (left, middle, right), in sterile 1 L glass flasks from 30 cm below the river surface. From each flask, duplicate volumes of 45 mL of river water were filled into sterile non-toxic 50 mL plastic vials (Techno Plastic Products AG, TPP, Trasadingen, Switzerland), containing 5 mL of glycerine (final conc. 10% v/v). The vials were completely mixed by hand and immediately stored at −20 °C on board of the cruise ship until analysis in the laboratory. After transfer to the laboratory (beginning in October 2013), the samples were stored at −80 °C. Fourteen sampling sites, four of them downstream of megacities (Vienna, Budapest, Belgrade and Bucharest), two at the beginning as well as two at the delta, four rural sampling sites, and two after confluence of two biggest tributaries (Drave, Tisa) were chosen for investigation (Table 1).

### 2.2. Isolation of Acinetobacter

The frozen samples were thawed, and 15 mL (left, middle, and right 5 mL each) were plated in 0.5 mL portions on selective agars. For the isolation of *Acinetobacter* 0.5 mL from left, middle, and right were plated on five agar-plates of CHROMagar™ (Oxoid, Germany) each. Growth conditions were 37 ± 1 °C for 18–24 h. Colonies were picked according to the manufacturer’s instructions and subcultured on Columbia blood-agar (in house production). Identification of *Acinetobacter* was carried out by matrix-assisted laser desorption ionization time-of-flight mass spectrometry (MALDI-TOF-MS) as described previously [18]. 

### 2.3. Susceptibility Testing

For inoculation, colonies were picked from an overnight pure culture on Colombia blood-agar (non-selective medium) with a sterile loop and suspended in sterile saline (0.85% NaCl w/v in water) to the density of a McFarland 0.5 standard (DensiCheck, Biomerieux, Vienna, Austria). The suspension was plated on Mueller-Hinton II agar using an automatic plate rotator (Retro C80, Biomerieux, Vienna, Austria). Antibiotic test disks were stamped on the agar surface. The plates were incubated at 36 °C for 16–20 h. After incubation, inhibition zones were determined. In case of testing susceptibility with Etest^®^, the same procedure for preparing the plates was carried out. Interpretation of zone-diameters and Etest^®^ was carried out according to the European Committee on Antimicrobial Susceptibility Testing (EUCAST) and if no EUCAST breakpoints were available Clinical Laboratory Standards Institute (CLSI) criteria were used for interpretation (Table 2) [19,20].

Etest for tigecycline was carried according to Altun et al. (2014) [21].

*Escherichia coli* ATCC 25922 and *Pseudomonas aeruginosa* ATCC 27853 were used as control strains in all performed tests.

### 2.4. Determination of β-Lactamase Genes

Determination of resistance genes was carried out for all *Acinetobacter* spp. isolates that revealed a resistance to at least one tested carbapenem. PCR detection and gene identification were performed for five different β-lactamases gene families, *bla*_CTX-M-1group_, *bla*_CTX-M-2group_, *bla*_CTX-M-9group_, *bla*_GES_, *bla*_KPC_, *bla*_OXA-23_, *bla*_OXA-24_, *bla*_OXA-51_, *bla*_OXA-48_, *bla*_OXA-58_, *bla*_NDM_, *bla*_SHV_, *bla*_TEM_, and *bla*_VIM._, PCR and sequencing procedures were performed as described previously [22,23,24,25,26,27].

## 3. Results

In total, 262 *Acinetobacter* were isolated. *Acinetobacter baumannii* was the most common species with 135 isolates. *Acinetobacter johnsonii* was second most with 62 isolates; all other species were represented by less than 20 isolates; *Acinetobacter haemolyticus* 19 isolates, *Acinetobacter junii* 17 isolates, *Acinetobacter lwoffii* 16 isolates, *Acinetobacter radioresistens* four, *Acinetobacter ursingii* two and seven isolates where no distinct species identification was possible. Non-*baumannii Acinetobacter* spp. were subsumed for further analyses.

**Figure 1:**
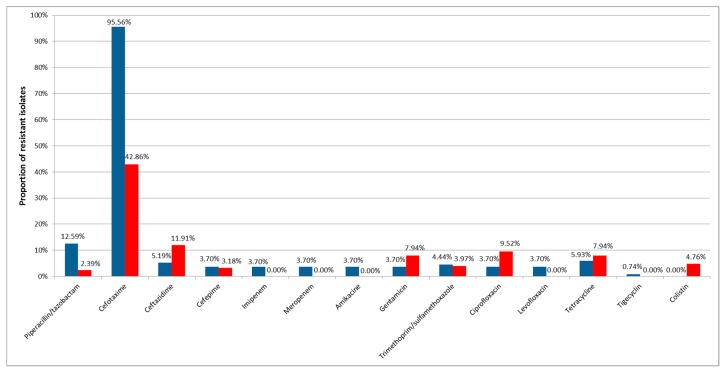
Percentage of resistance to tested antibiotics of isolated *Acinetobacter baumannii* (blue) and non*-baumannii Acinetobacter* spp. (red).

Susceptibility testing revealed that resistance to the most tested antibiotics was rare in *Acinetobacter baumannii* and non*-baumannii Acinetobacter* spp. river water isolates. The only resistance present in both groups in more than 10% of the isolates was to cefotaxime with 95.6% (129/135) of *Acinetobacter baumannii* and 43.3% (55/127) non*-baumannii Acinetobacter* spp. In addition, resistances to ceftazidime in non*-baumannii* (12.6%, 16/127) and to piperacillin/tazobactam in *Acinetobacter baumannii* (12.6%, 17/125) were present in more than 10% in one of the sample subgroups (Figure 1).

Resistance to fluoroquinolones showed one notable detail: all five *Acinetobacter baumannii* were resistant to ciprofloxacin and levofloxacin, whereas non-*baumannii Acinetobacter* spp. remained susceptible to the second tested fluoroquinolone. Less than 10 *Acinetobacter baumannii* isolates revealed resistance to all other tested antibiotics, all isolates were susceptible to colistin. In contrast to this, colistin resistance could be detected in six (4.7%) non*-baumannii Acinetobacter* spp., and no resistance was found to levofloxacin, imipenem, meropenem, amikacine, and tigecycline (Figure 1).

Only six (4.4%) *Acinetobacter baumannii* revealed susceptibility to all tested antibiotics, but only 16 (11.9%) were resistant to one or more tested antibiotics additionally to cefotaxime. Six (4.4%) isolates could be classified as multiresistant, with resistance to antibiotics from at least three different antibiotic classes, including one isolate only susceptible to colistin and four to colistin and tigecyline (Table 3).

Susceptibility to all antibiotics was 10 times higher (55 isolates, 43.3%) in the non*-baumannii Acinetobacter* spp. group compared to the *Acinetobacter baumannii group*. Only 10 isolates revealed multiresistance. Two isolates with different resistance profile showed resistance to four antibiotics (JDS10AC012 to CTX, piperacillin/tazobactam, FEP, and ceftazidime; JDS38AC048 to CTX, TZP SXT and CAZ).

Five *Acinetobacter baumannii* were resistant to carbapenems (meropenem and imipenem). These isolates were analyzed for the presence of several β-lactamases genes, resulting in four different gene patterns. Classic intrinsic OXA carbapenemases were present in all isolates, but no gene was present in all isolates. JDS59AC007 and JDS59AC001 were positive for OXA-23 and OXA-51, and JDS38AC020 was positive for OXA-24 and OXA-51. In addition to OXA-23 and OXA-51, two isolates revealed β-lactamases from another Ambler class: JDS38AC018 and JDS38AC017 harbored both the gene for carbapenemase VIM-2 and JDS38AC017 harbored additionally the gene for the broad spectrum β-lactamases TEM-1 (Table 3).

## 4. Discussion

The presence of *Acinetobacter* with human-induced multidrug resistance phenotypes in surface water has been reported from all over the world. Their origin seems to be influenced by (treated and untreated) hospital waste water. The impact (proportion and persistence) of these strains on the *Acinetobacter* water population is not well documented [9,14,15,28]. This study shows for the first time the susceptibility phenotypes of *Acinetobacter* of a total European river system. Furthermore, our study provides a first glimpse of the anthropogenic impact on the *Acinetobacter* river population. In the *Acinetobacter* population of the River Danube, even resistance to last line antibiotics (e.g., colistin and tigecycline) is detectable, and this without using selective media by screening only a relatively small volume of water. This screening led to the detection of multidrug-resistant *Acinetobacter baumannii* isolates, whose multidrug resistance would normally be found and related only to intensive care units. The isolation of multiresistant *Acinetobacter* was limited to the area of influence of megacities, but even there the great majority of isolates remained not or only slightly influenced on their susceptibility pattern.

Looking at the resistance data for invasive *Acinetobacter* isolates for the Danube neighboring countries reveals a rather gloomy picture: In clinical isolates, the ratio for carbapenem resistance spans from 5.5% in Germany to over 80% in Romania. In our study of the river water, however, only five isolates (less than 2% of all isolates) showed resistance to carbapenems, a very low proportion of resistant *Acinetobacter* spp. compared with the clinical settings [29]. This ratio corresponds with our findings in the *Pseudomonas* population in the river Danube, where we also found a ratio of around 2% [30,31].

In a study of the Jadro River, carried out by Maravic et al., only selected multi-drug-resistant *Acinetobacter* were isolated (using selective media with supplements). Comparing these isolates with the Danube isolates, there is a remediable difference as regards aminoglycoside resistance. Maravic et al. did not detect a single isolate that was resistant to the tested aminoglycoside in contrast to 5.7% (15 isolates) from the River Danube. Furthermore, only two multiresistant Danube isolates revealed no resistance to one of the tested aminoglycoside. Carbapenem resistance in Jadro River isolates was restricted to meropenem, but the number of isolates was too low to be noteworthy. Interestingly enough, the proportion of cefotaxime resistance is nearly identical with 68% in Jadro River and 66% in the river Danube [9].

## 5. Conclusions

Multiresistant strains can be found in our environment, in any habitat and at any time, making chances for contact high and permanent. Further investigation will show if the spread of multiresistant *Acinetobacter* has reached its peak and if susceptible environmental *Acinetobacter* will still outnumber the clinical strains or if we have to further deal with a constant increase of non-susceptible *Acinetobacter* in the future.

## Figures and Tables

**Table 1:** JDS3 sampling sites chosen for isolation and their assignment to the upper-, middle-, or downstream stretches (SP = sampling point; us = upstream; ds = downstream). Country codes: Germany: DE; Austria: AT; Hungary: HU; Croatia: HR; Serbia: RS; Romania: RO; Bulgaria: BG.

SP	Name of SP	River (km)	Country
JDS2	Kelheim, gauging station	2415	DE
JDS3	Geisling power plant	2354	DE
JDS8	Oberloiben	2008	AT
JDS10	Wildungsmauer (Vienna)	1895	AT
JDS22	ds Budapest	1632	HU
JDS28	us Drava	1384	HR/RS
JDS36	ds Tisa/us Sava	1200	RS
JDS38	us Pancevo (Belgrade)	1159	RS
JDS49	Pristol/Novo Salo	834	RO/BG
JDS57	ds Ruse	488	RO/BG
JDS59	ds Arges (Bucharest)	429	RO/BG
JDS63	Siret	154	RO
JDS67	Sulina Arm	26	RO
JDS68	St. Gheorge Arm	104	RO

**Table 2:** List of tested antibiotics, concentration on the disc (Sensi-DiscTM paper discs, BD, Vienna, Austria) or Etest^®^ (Biomerieux) and antibiotic classes.

Antibiotic	Concentration	Antibioti Classes
piperacillin/tazobactam	100 µg/10 µg	β-lactam
cefotaxime	30 µg	β-lactam
ceftazidime	30 µg	β-lactam
cefepime	30 µg	β-lactam
imipenem	10 µg	β-lactam
meropenem	10 µg	β-lactam
amikacin	30 µg	aminoglycoside
gentamicin	10 µg	aminoglycoside
trimethoprim/sulfamethoxazole	1.25 µg/23.75 µg	folate synthesis inhibitors
ciprofloxacin	5 µg	quinolone
levofloxacin	5 µg	quinolone
tigecycline	Etest	tetracyclin
tetracycline	30 µg	tetracycline
colistin	Etest	polypeptide antibiotic

**Table 3:** Detected resistance genes in carbapenem resistant *Acinetobacter baumannii*; us = upstream; ds = downstream.

Isolate	Site of Isolation	Susceptible Antibiotics	Detected β-Lactamases
JDS38AC017	us Pancevo (Belgrade)	colistin, tigecycline	OXA-23, OXA-51, VIM-2
JDS38AC018	us Pancevo (Belgrade)	colistin	OXA-23, OXA-51, VIM-2, TEM-1
JDS38AC020	us Pancevo (Belgrade)	colistin, tigecycline	OXA-24, OXA-51,
JDS59AC001	ds Arges (Bucharest)	colistin, tigecycline	OXA-23, OXA-51
JDS59AC007	ds Arges (Bucharest)	colistin, tigecycline	OXA-23, OXA-51
